# Elevated iron concentration in putamen and cortical speech motor network in developmental stuttering

**DOI:** 10.1093/brain/awab283

**Published:** 2021-11-09

**Authors:** Gabriel J Cler, Saloni Krishnan, Daniel Papp, Charlotte E E Wiltshire, Jennifer Chesters, Kate E Watkins

**Affiliations:** 1 Wellcome Centre for Integrative Neuroimaging, Department of Experimental Psychology, University of Oxford, Oxford OX2 6GG, UK; 2 Department of Psychology, Royal Holloway, University of London, Egham Hill, Surrey TW20 0EX, UK; 3 Wellcome Centre for Integrative Neuroimaging, FMRIB Centre, Nuffield Department of Clinical Neuroscience, University of Oxford, Oxford OX3 9DU, UK; 4 Bristol Speech and Language Therapy Research Unit, North Bristol NHS Trust, Bristol BS10 5NB, UK

**Keywords:** developmental stuttering, basal ganglia, iron, quantitative imaging

## Abstract

Theoretical accounts of developmental stuttering implicate dysfunctional cortico-striatal-thalamo-cortical motor loops through the putamen. However, the analysis of conventional MRI brain scans in individuals who stutter has failed to yield strong support for this theory in terms of reliable differences in the structure or function of the basal ganglia.

Here, we performed quantitative mapping of brain tissue, which can be used to measure iron content alongside markers sensitive to myelin and thereby offers particular sensitivity to the measurement of iron-rich structures such as the basal ganglia.

Analysis of these quantitative maps in 41 men and women who stutter and 32 individuals who are typically fluent revealed significant group differences in maps of R_2_*, indicative of higher iron content in individuals who stutter in the left putamen and in left hemisphere cortical regions important for speech motor control. Higher iron levels in brain tissue in individuals who stutter could reflect elevated dopamine levels or lysosomal dysfunction, both of which are implicated in stuttering.

This study represents the first use of these quantitative measures in developmental stuttering and provides new evidence of microstructural differences in the basal ganglia and connected frontal cortical regions.

## Teaser

Using quantitative MRI brain mapping, Cler *et al*. reveal higher levels of iron in brain regions relevant to speech motor control in adults who stutter. Higher levels of iron may indicate elevated dopamine levels or lysosomal dysfunction, providing evidence to support theoretical accounts of stuttering.

**See Sommer *et al.* (doi:10.1093/brain/awab348) for a scientific commentary on this article.**

## Introduction

Developmental stuttering is characterized by dysfluent speech and is observed in 8% of children and ∼1% of the general population.^1^ Theoretical accounts of developmental stuttering implicate dysfunctional cortico-basal ganglia-thalamocortical motor circuits through the putamen.^2^^,^^3^ Supportive evidence for this theory comes from observations of shared motor characteristics with basal ganglia disorders, such as Parkinson’s disease and dystonia, as well as changes to fluency in response to dopaminergic medication or to deep brain stimulation.^2^ Early PET studies indicated abnormal basal ganglia function during speech production and differences in dopamine metabolism in individuals who stutter.^4^^,^^5^ However, recent meta-analyses that included functional MRI studies failed to identify dysfunction in these regions as either a state or trait characteristic of stuttering.^6^ In terms of structure, conventional MRI scans have been used in individuals who stutter to measure grey matter volume, cortical thickness, and diffusion properties of white matter fibre tracts. Whereas some consensus on the occurrence of white matter abnormalities has been reached, analysis of cortical and subcortical volumes reveals inconsistent findings of grey matter differences in individuals who stutter.^6^^,^^7^ In sum, even though there is strong theoretical evidence that points to basal ganglia dysfunction in developmental stuttering, imaging evidence to support or refute this theory is lacking.

In the current study, we scanned the brains of a large sample of individuals who stutter and a group of age- and gender-matched individuals who are typically fluent using a multi-parameter mapping (MPM)^8^ protocol, which produces semi-quantitative whole-brain maps of three parameters: R_1_, MTsat, and R_2_* (longitudinal relaxation, magnetization transfer saturation, and effective transverse relaxation rate, equivalent to 1/T_2_*, respectively). These parameters reflect histologically-verified differences in tissue microstructure related to myelin and iron.^8^^,^^9^ R_1_ and MTsat correlate with the amount of myelin in grey and white matter,^9^ and R_2_* correlates with post-mortem estimates of iron deposits in grey matter.^10^

Iron is found in greatest concentration in the basal ganglia. Too little iron is considered detrimental during early development, and lower R_2_* in the basal ganglia is associated with poorer cognitive ability in adolescents.^11^ On the other hand, greater iron concentration, as indicated by higher R_2_*, is a hallmark of Parkinson’s disease^12^ and increases with ageing.^13^ We predicted, therefore, that this parameter would be sensitive to any previously undetected differences in the basal ganglia in individuals who stutter, but we did not have a directional hypothesis about whether R_2_* (iron concentration) would be higher or lower relative to individuals who are typically fluent.

## Materials and methods

### Participants

Multi-parameter maps were acquired from 73 participants: 41 individuals who stutter (nine women) and 32 individuals who are typically fluent (nine women). Data from an additional 18 participants were excluded because of: acquisition error related to head placement (two individuals who stutter, one individual who is typically fluent); low image or map quality due to movement (eight individuals who stutter; seven individuals who are typically fluent; Supplementary material). Individuals who stutter were aged 19–45 years (median: 31.2), and individuals who are typically fluent were aged 19–44 years (median: 28.6); the groups did not significantly differ in age [*t*(64.4) = −1.5, *P *>* *0.14]. Stuttering severity was assessed in individuals who stutter with the SSI-4 (Stuttering Severity Instrument-Fourth Edition^14^). The men who stutter were scanned as part of a baseline session of a treatment study that required them to have at least a mild/moderate score at screening (SSI ≥ 20). All participants who stutter met this criterion, but some had milder scores when retested during the baseline session, including one who was classed as very mild (SSI = 16). Thus, the final scores ranged from 16 to 40 (median: 25). Participants had no history of speech, language, or neurological conditions other than developmental stuttering. Participants provided written consent and were compensated for their participation. All procedures were approved by the University of Oxford ethics committee.

### Image acquisition

Scans were acquired on a Siemens Prisma 3T scanner with three 3D multi-echo FLASH scans of predominantly T_1_ (T_1_w), proton density (PDw), and magnetization transfer weighting (MTw), along with a B1 transmit field map and a B0 static field map.^8^ A tailored pulse sequence was used: 1 × 1 × 1 mm resolution, field of view = 256 × 224 × 176 mm^3^, repetition time = 25 ms, bandwidth = 488 Hz/pixels, first echo time/echo spacing = 2.3/2.3 ms, 6° flip angle (PDw, MTw), or 21° (T_1_w), slab rotation = 30°, and number of echoes = 8 (PDw, MTw) or 6 (T_1_w), GRAPPA acceleration factor 2 × 2, 40 reference lines in each phase-encoded direction. B1 maps were acquired with spin echo and stimulated echo with a repetition time of 500 ms, a spin-echo echo time of 37.06 ms and a mixing time of 33.8 ms. B0 maps were acquired with repetition time of 1020 ms and echo times of 10 and 12.46 ms.

### Quantitative map estimation

**Figure 1:**
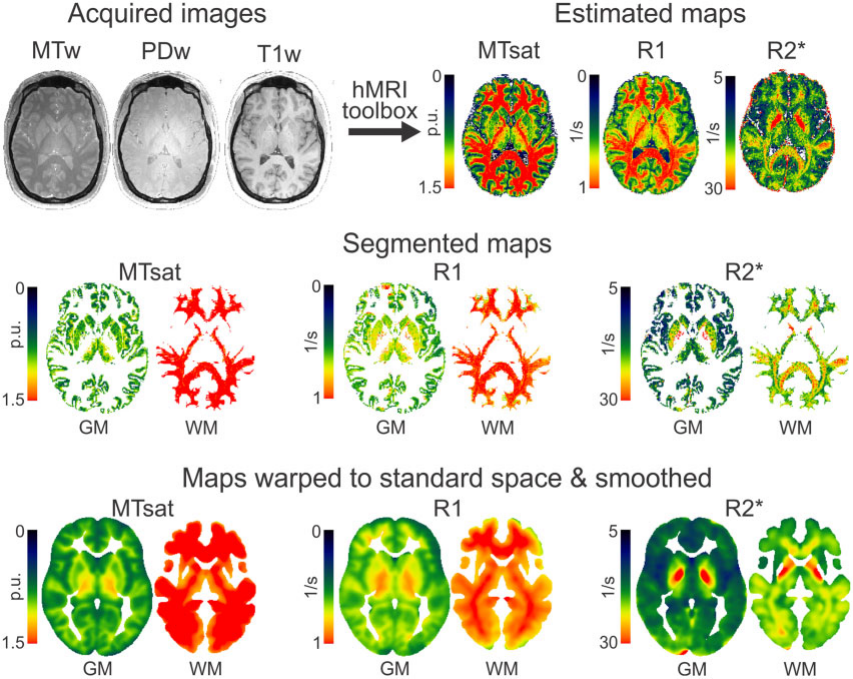
**Schematic of processing pipeline.** Data from one participant shown through each stage of processing, with acquisition [proton density (PDw), magnetization transfer (MTw), and T_1_-weighted (T1w) images in native space] followed by processing in the hMRI toolbox running in SPM: map estimation (using PDw, MTw, and T1w images to calculate MTsat, R_1_, and R_2_* maps), map segmentation into separate grey and white matter tissue (calculated from MTsat and applied to all maps), warping maps to standard space (calculated from MTsat and applied to all maps), and smoothing. R_1_ and R_2_* values are in units of 1/s; MTsat are per cent units (p.u.). Colour maps are scaled per parameter to show variation in grey matter. GM = grey matter; WM = white matter.

The hMRI toolbox was used to calculate and process parameter maps of R_1_, MTsat, and R_2_* from the proton density, magnetization transfer, and T_1_-weighted images using the integrated pipeline with default settings.^9^ Maps were segmented into grey and white matter and registered to MNI space. Segmented maps were smoothed with a 6-mm Gaussian full-width at half-maximum (sigma = 2.55). Importantly, all processing preserved the quantitative parameter values, without modulating for volume changes.^9^ A schematic with images and maps from these steps for a single participant is shown in Fig. 1.

### Statistical analysis

Statistical analysis was performed using the FMRIB Software Library (FSL). For each map and tissue type (MTsat, R_1_, R_2_* × white, grey matter), a whole-brain general linear model analysis was performed using permutation testing with 5000 permutations. Statistical inference was drawn using threshold-free cluster enhancement (TFCE) to identify voxels in which the measurements between groups differed significantly. TFCE identifies regions of statistical difference without specifying an arbitrary cluster-forming height threshold; instead, small regions that are very different between groups or large regions with smaller differences may be identified, as long as they exhibit cluster-like regional specificity.^15^ Significance was set at *P* < 0.025 to correct for the two single-tailed *t*-tests performed to assess group differences in both directions.^16^

The remaining statistical analyses were performed in R using analyses of variance (R version: 4.0.2; command: aov). Mean values of R_2_* were extracted for the statistical clusters in regions showing significant group differences to test relationships between R_2_* and age and compare these relationships between groups (age, group, and age × group as factors). Within the individuals who stutter group, the relationship to stuttering severity in these regions was tested statistically with R_2_* as the dependent measure and age, stuttering severity index, and age × stuttering severity as factors.

### Data availability

Statistical maps of group differences are available at NeuroVault: https://neurovault.org/collections/UPUDTUZJ/.

## Results

Individuals who stutter and those who are typically fluent did not differ in terms of whole brain volume or values averaged across all voxels in the grey and white tissue maps of R_1_, MTsat and R_2_* (Supplementary Table 1).

**Figure 2:**
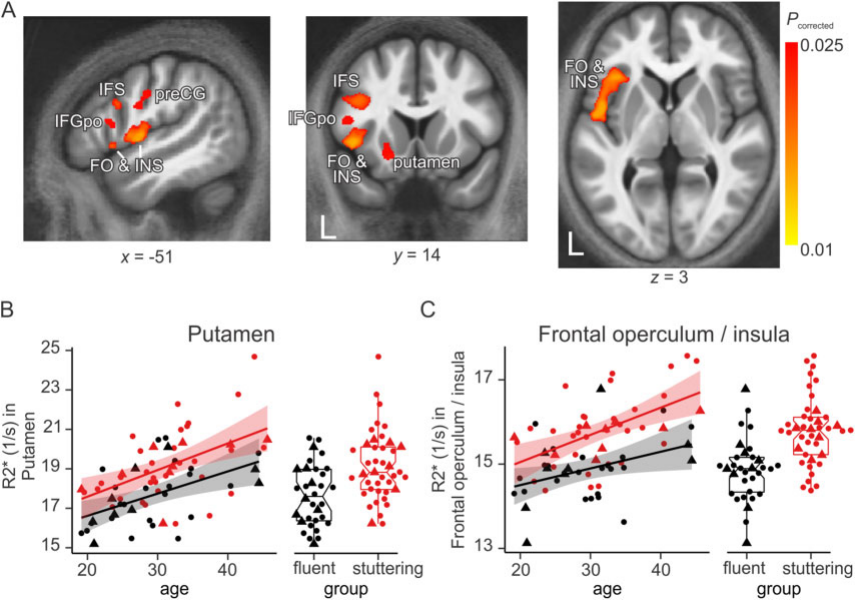
Areas with higher R_2_* in individuals who stutter. (**A**) The coloured overlay is the statistical map showing areas with higher R_2_* in individuals who stutter than individuals who are typically fluent (thresholded at *P* < 0.025) on top of the average MTsat map for all participants aligned to MNI space. Individual data for mean R_2_* are plotted against age × group in (**B**) left putamen, (**C**) left frontal operculum and insula. Red = individuals who stutter; black = individuals who are typically fluent. Circles are men and triangles are women. Shaded areas show 95% confidence intervals. In box plots, centre line is median and edges are 25th and 75th percentiles. L = left hemisphere; preCG = precentral gyrus; IFS = inferior frontal sulcus (posterior); IFGpo = inferior frontal gyrus, pars opercularis; FO & INS = frontal operculum/insula.

Whole-brain voxel-wise analysis of the quantitative maps revealed significantly higher grey matter R_2_* in individuals who stutter compared with individuals who are typically fluent subcortically in the left putamen, and cortically primarily in the left frontal lobe, including: the frontal opercular cortex extending to the anterior insula; the inferior frontal gyrus (pars opercularis) and posterior extent of the inferior frontal sulcus; and ventral precentral gyrus corresponding to the level of the representation of tongue movements^17^ (Table 1 and Fig. 2A). It was striking that at this threshold (*P* < 0.025), the group differences were restricted to the left hemisphere. At a lower threshold of *P* < 0.05, group differences were also seen in the left caudate nucleus, subcortically, and more extensive portions of the right and left hemispheres, cortically (Supplementary Fig. 1).

**Table 1:** Locations of increased R_2_* in individuals who stutter

Brain area	*x*	*y*	*z*	Voxels	*P*
Left superior frontal sulcus	−24	27	40	513	0.023
Left inferior frontal gyrus, pars opercularis	−49	15	14	229	0.024
Left inferior frontal sulcus (posterior)	−42	11	27	1677	0.019
Left putamen	−21	10	−11	615	0.023
Left frontal operculum/anterior insula	−46	8	3	6674	0.012
Left precentral gyrus (ventral)	−52	−6	29	499	0.024
Left planum temporale	−56	−30	15	6	0.025
Left superior parietal lobule	−28	−60	56	1626	0.018

Thresholded at *P* < 0.025. Coordinates of the centre of gravity of each cluster are provided in MNI152 space. The extent of each cluster is provided in voxels. The *P*-value for the peak voxel is shown in the final column.

Mean R_2_* values for each significant cluster are shown for each participant (Fig. 2B, C and Supplementary Table 2) and examined further against age (Supplementary Fig. 2). As expected,^13^ there was a significant linear increase in R_2_* with age in all regions, but importantly these relationships did not differ between groups (i.e. no interaction between group and age; Supplementary Table 2).

To check that the focal differences in grey matter R_2_* were not due to morphometric differences in individuals who stutter, we performed a voxel-based morphometry (VBM) analysis using standard T_1_-weighted images (Supplementary material). The groups were not different in terms of their relative amounts of grey matter anywhere in the brain.

In the individuals who stutter, we examined if there was a significant relationship between stuttering severity and R_2_* when controlling for age in any of the grey matter regions showing higher R_2_*; no regions showed a significant relationship between R_2_* and stuttering severity (Supplementary Table 3).

There were no differences between groups in the white matter R_2_* maps or in the grey or white matter maps of the myelin-sensitive markers, R_1_ and MTsat.

## Discussion

Obtaining multi-parameter maps in a large cohort of individuals who do and do not stutter allowed us to conduct a detailed examination of neural microstructure, tied to histologically- and neurobiologically-relevant processes. Our results provide evidence of microstructural differences in those who stutter consistent with theoretical accounts of developmental stuttering that implicate dysfunction in cortico-basal ganglia-thalamocortical loops through the putamen.^2^^,^^3^ Below we discuss these findings in the context of the relationships between iron in brain tissue and dopamine. We also discuss the link to lysosomal dysfunction, which is implicated in stuttering through the identification of causative mutations in related genes.

### R_2_* differences in the putamen and cortical speech motor regions

The R_2_* parameter provided by the quantitative mapping protocol has known sensitivity to non-heme iron (that is, iron in the tissue rather than in blood), based on direct comparisons of R_2_* to histology in post-mortem brains with and without neurodegenerative diseases.^10^^,^^18^ Iron in the brain is found in highest concentration in the basal ganglia.^13^ For this reason, we predicted it would be sensitive to detecting differences in these nuclei in individuals who stutter. Accordingly, one region in the basal ganglia had higher R_2_* in individuals who stutter: the left putamen, which has previously been implicated in theoretical accounts of stuttering.^2^^,^^3^ Wu and colleagues^4^ found increased dopaminergic activity in the left insula and putamen in a very small sample of individuals who stutter. Another PET study showed that treatment success in individuals who stutter was predicted by decreased regional cortical blood flow in the left putamen.^19^

In addition to this basal ganglia difference, there were several cortical regions showing higher R_2_* in individuals who stutter (Table 1), all of which are part of the speech motor network. These left hemisphere cortical areas in inferior frontal and ventral motor cortex show disfluency-related activity and are commonly underactive in individuals who stutter relative to controls (state and trait).^6^ How the R_2_* differences in these brain regions relate to abnormal patterns of brain activity or to stuttering and other motor characteristics in individuals who stutter is as yet unknown. Our analyses failed to reveal relationships in any area with a standardized measure of stuttering severity.

### Possible interpretations of higher R_2_*/iron concentration: dopamine and lysosomal dysfunction

One explanatory model for higher iron concentration in grey matter in individuals who stutter implicates dopamine. Iron and dopamine have complex interactions in the brain and must remain in a precise homeostatic balance for healthy function. Accordingly, increasing extracellular dopamine leads to higher intracellular iron levels, and when iron is introduced in the cell, D2 receptor protein concentrations increase.^20^^,^^21^ A recent study in typically fluent speakers has suggested R_2_* as a correlate of presynaptic vesicular dopamine concentration.^22^ Thus R_2_* increases seen here in individuals who stutter could be an indirect marker of excess dopamine levels. Developmental stuttering has been hypothesized to result from an excess of dopamine, although the evidence in support of this hypothesis from PET and pharmacological interventions is weak due to small sample sizes or side effects of the medication.^2^^,^^4^^,^^23^ On the other hand, R_2_* is also increased in Parkinson’s disease in which dopamine is depleted; in that case, it is thought that the increased iron may be the causative agent that leads to the death of dopaminergic neurons in the substantia nigra.^24^ This mechanism is related to lysosomal dysfunction, which is also implicated in stuttering and forms the basis of our second explanatory model for the increased iron concentration in individuals who stutter.

In typical function, lysosomes degrade and recycle cellular waste, repair plasma membranes, and decompose intracellular stores of ferritin (Fe^3+^) into Fe^2+^ to be transported for cellular processes requiring iron ions.^25^ Accordingly, lysosomal storage disorders can result in an accumulation of substrates that are otherwise typically decomposed or transported. One such disorder, Gaucher disease, leads to an accumulation of iron in the brain and body. The genetic mutation that causes Gaucher disease is homozygous; when that same mutation is heterozygous, individuals have an increased risk of developing Parkinson’s disease.^26^ Heterozygous causative mutations in *GNPTAB*, *GNPTG*, and related genes have been identified in individuals who stutter, accounting for ∼10% of these individuals.^27^ Homozygous mutations in these genes result in dysfunctions in intracellular trafficking and lysosomal processes.^27^ Expression patterns of these genes in the Allen Human Brain Atlas are spatially coincident with the cortical networks showing differences in children who stutter and are particularly high in the frontal opercular cortex, where we found significantly higher concentrations of iron in adults who stutter.^28^ We therefore posit that the increased iron could be indicative of lysosomal dysfunction. We found that R_2_* increased with age, as expected,^13^ but increases were not accelerated in individuals who stutter (no significant interaction between age and group; Supplementary Table 2). Thus, it is possible that heterozygous lysosomal mutations in stuttering cause dysfunction at a critical period in development or lead to lysosomal dysfunction resulting in greater iron deposition that does not accumulate at a faster rate over time.

### No differences were found in myelin markers or grey matter volume

Despite our reasonably large sample size, our analysis did not detect group differences in the other parameters provided by the quantitative maps or in grey matter volume. No differences were found in MTsat or R_1_ maps, both of which are thought to be sensitive to differences in the amount of myelin in a given area.^9^ This result may appear inconsistent with the results of many diffusion weighted imaging studies in individuals who stutter (including our own), which reported lower fractional anisotropy (FA) in white matter tracts.^7^ Lower FA could reflect a number of differences in white matter microstructure, including the orientation or dispersion of fibres in a voxel, axonal calibre and density, and not only the amount of myelin. We speculated previously that lower FA in individuals who stutter in some areas reflected differences in fibre organization, rather than amount of myelin.^29^ The current results are consistent with that interpretation. Nevertheless, further studies are warranted to understand the relationships among these different measures of microstructure.

VBM has previously been used to examine whether regional amounts of grey matter differ in individuals who stutter relative to control groups.^7^ The findings are equivocal: in some studies, individuals who stutter have more grey matter, in others less, and in others no group differences are reported. We found no significant differences in amounts of grey matter in any brain region, and thus find no evidence that the R_2_* differences are related to differing amounts of grey matter in individuals who stutter.

This is the first study using multi-parameter mapping to measure R_2_*, R_1_, or MTsat in individuals who stutter. Even though it is in quite a large cohort (41 individuals who stutter, 32 individuals who are typically fluent), these novel results need to be replicated. One recent study has indicated similar findings in regard to iron using ultrasound to reveal elevated iron accumulation in the substantia nigra in individuals who stutter.^30^ Here we similarly conclude that there are iron differences in the basal ganglia (and connected cortical areas) in individuals who stutter, although our analyses revealed no significant differences in iron levels in the substantia nigra. Further analyses are warranted and measurement using ultrasound in the same participants would clarify the current discrepancy between the results of the two studies.

## Conclusion

In this study of a large sample of individuals who stutter, we provide evidence for elevated R_2_* in the left putamen and connected frontal cortical regions. This difference in R_2_* most likely reflects increased iron concentrations, which may be indicative of excess dopamine levels or lysosomal dysfunction in individuals who stutter. Further work is needed to link R_2_* differences to genetic profiles associated with developmental stuttering, to increased dopamine or lysosomal dysfunction, or to another neurobiological function that could point towards effective therapies for those who want them.

## Supplementary Material

awab283_Supplementary_Material

## Abbreviations

MTsat: magnetization transfer saturation
