# Supplementary material for: Elevated iron concentration in putamen and cortical speech motor network in developmental stuttering
Source: Brain. 2021 Nov 9;144(10):2979–84. doi: 10.1093/brain/awab283 (PMC8634076; doi:10.1093/brain/awab283)
Supplement: awab283_Supplementary_Material [file awab283_supplementary_material.pdf]

Supplementary material

for Cler, Krishnan, Papp, Wiltshire, Chesters, & Watkins (2021) - Elevated iron concentration in putamen and cortical speech motor network in developmental stuttering

Table of Contents:

Map quality assessment (Methods)

VBM Analysis (Methods & Results)

Figures

Supplemental Figure 1. Subthreshold areas with higher R2* in individuals who stutter.

Supplemental Figure 2. Age and group effects on R2*

Supplemental Figure 3. Results of semi-automated quality control algorithm

Tables

Supplemental Table 1. Parameters for each tissue type across the whole brain.

Supplemental Table 2. Effects of age on R2* in statistical clusters; no interaction between age and group

Supplemental Table 3. No effect of severity on R2* in individuals who stutter

References

Map quality assessment (Methods)

Map quality was assessed using visual inspection and quantitative quality measures. All maps from 91 participants were visually inspected and images with acquisition errors were excluded (2 individuals who stutter, 1 individual who is typically fluent). We calculated the coefficient of variation (CoV) within the segmented white matter and grey matter quantitative maps.[1] Visual inspection of the data distribution of the CoV within the R1 map revealed an obvious cutoff at 0.18 (see Supplemental Figure 3). Data for all maps from participants with R1 CoV values above this threshold were removed (8 individuals who stutter; 7 individuals who are typically fluent). The hMRI toolbox also automatically provides quality assessment measures representing motion during the initial scans;[2] the CoV measure is an assessment of the quality of the final parameter maps. For inter-scan motion, total translation was calculated as the Euclidean distance from the origin to the coregistration X,Y,Z (in mm) provided by the toolbox of MTw to PDw; coregistration T1w to PDw. For intra-scan motion, we compared standard deviation in white matter of the R2* maps (SD-R2*), calculated from each individual multi-echo acquisition (PDw, T1w, MTw). These are differentiated from the final R2* maps used in the statistical analyses, as those are generated from all three acquisitions and then combined via ESTATICS.[2] Data from all participants who were outliers on these five inter- and intra-scan motion measures (by visual inspection and statistically >3.5 SD from the mean on any of five automated measures: coregistration MTw to PDw; coregistration T1w to PDw; SD-R2* from MTw; SD-R2* from PDw; SD-R2* from T1w) were already excluded based on the CoV measure. Thus, data from the remaining 73 participants were included in all further analyses.

VBM Analysis (Methods & Results)

To ensure that quantitative differences in parameter maps of grey matter were not due to morphometric group differences, we also performed a voxel-based morphometry (VBM) analysis using standard MPRAGE images. High-resolution T1-weighted structural images were acquired alongside the quantitative parameter acquisitions with an MPRAGE protocol (PAT2, 1mm isotropic, TR/TE = 2400/3.98). There were 38 individuals who stutter and 27 individuals who are typically fluent in this analysis, as there were no standard MPRAGE images acquired in the first eight participants. Images were analysed with FSL-VBM, wherein images were brain-extracted, grey matter-segmented, and registered to MNI152 standard space using non-linear registration. The resulting images were averaged and flipped along the x-axis to create a left-right symmetric, study-specific grey matter template using an equal number of individuals who stutter and individuals who are typically fluent (27 participants who stutter were selected at random and combined with data from 27 individuals who are typically fluent). All original grey matter images were non-linearly registered to this study-specific template and modulated to correct for local expansion or contraction due to the non-linear component of the spatial transformation. The modulated grey matter images were then smoothed with an isotropic Gaussian kernel with a sigma of 3 (~7mm FWHM). Images were statistically analysed identically to the parameter maps: a voxel-wise general linear model was applied using permutation-based non-parametric testing, correcting for multiple comparisons across space. Statistical inference was drawn using threshold-free cluster enhancement at $p<.05$ to identify voxels in which the measurements between groups differed significantly in either direction.

No significant differences were found when correcting for multiple comparisons across space. Since the purpose of our VBM analysis was to confirm that the quantitative differences in R2* were not explained by morphometric differences in the cortex or subcortical grey matter, we lowered the threshold to uncorrected $p<.05$ ($t>2.1$) in the regions with elevated R2* in individuals who stutter. While none of the regions with sub-threshold VBM differences overlapped those with R2* differences, two regions of nearby cortex showed morphometric differences: (i) individuals who stutter had more grey matter in the most ventral extent of the post-central sulcus, just dorsal to the portion of central opercular cortex with higher R2* (MNI: [-56 -19 23], t=3.95, $p_{uncorr}<.0006$); (ii) individuals who stutter had more grey matter in cortex adjacent and posterior to the portion of inferior frontal sulcus with higher R2* (MNI: [-46 2 30], t=3.48, $p_{uncorr}<.002$). We conclude that differences in R2* are not explained by or overlapping with differences in the amount of grey matter.

Figures

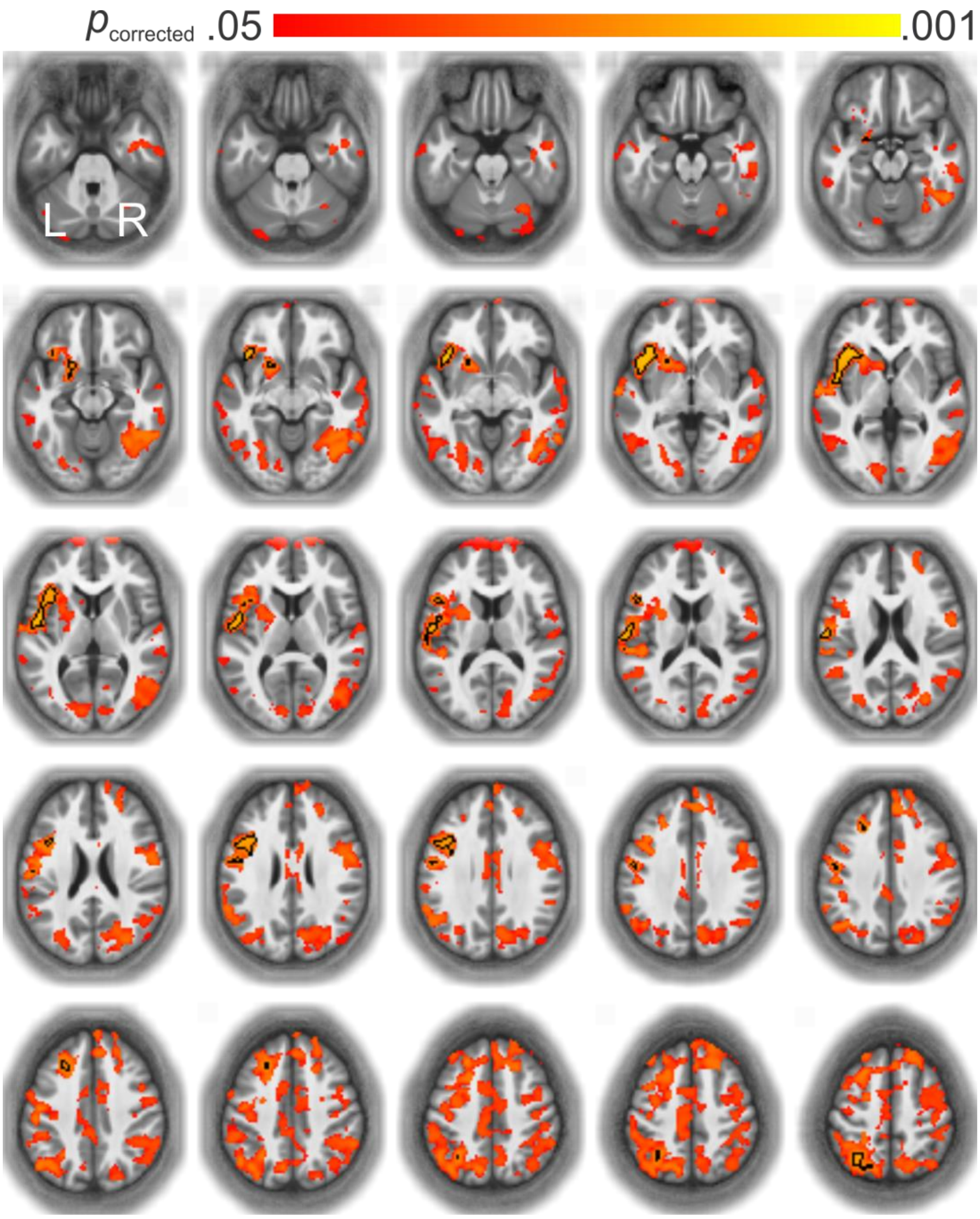

Supplemental Figure 1. Subthreshold areas with higher R2* in individuals who stutter. Coloured overlay is the statistical map showing areas with higher R2* in individuals who stutter than individuals who are typically fluent; thresholded at corrected $p<.05$. Statistically significant areas at a corrected threshold of $p<.025$ are outlined in black. Shown on top of the average MTsat map for all participants aligned to MNI space. L: left; R: right hemispheres.

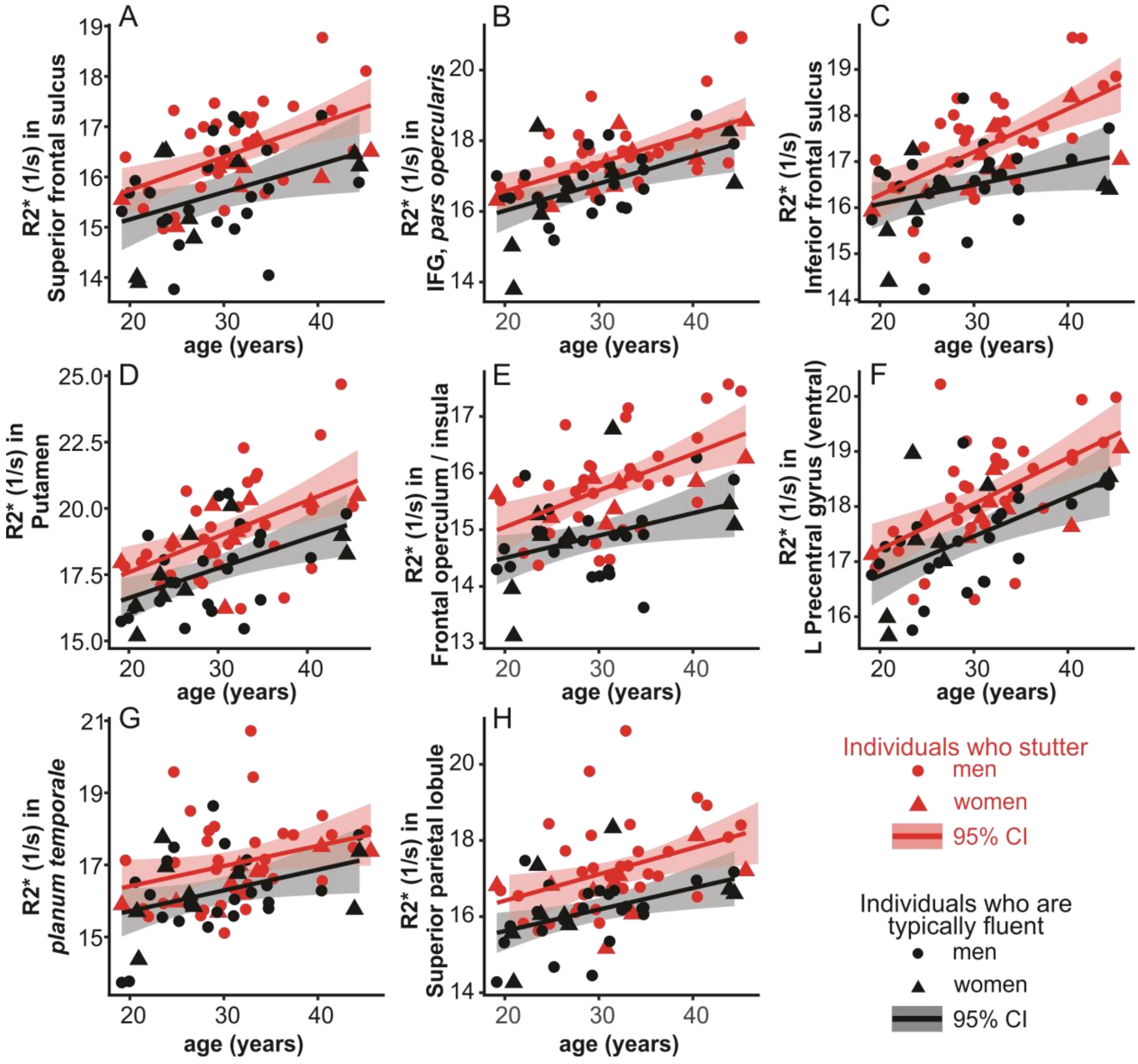

Supplemental Figure 2. Age and group effects on R2* in all areas with higher R2* in individuals who stutter ($p < .025$). Associated statistical results are shown in Supplemental Table 1. Red are individuals who stutter; black are individuals who are typically fluent. Circles are men and triangles are women. Shaded areas show 95% confidence intervals. **(A)** L Superior frontal sulcus; **(B)** L Inferior frontal gyrus, pars opercularis; **(C)** L Inferior frontal sulcus (posterior); **(D)** L Putamen (same as Figure 2D); **(E)** L Frontal operculum / insula (same as Figure 2E); **(F)** L Precentral gyrus (ventral); **(G)** L planum temporale; **(H)** L Superior parietal lobule.

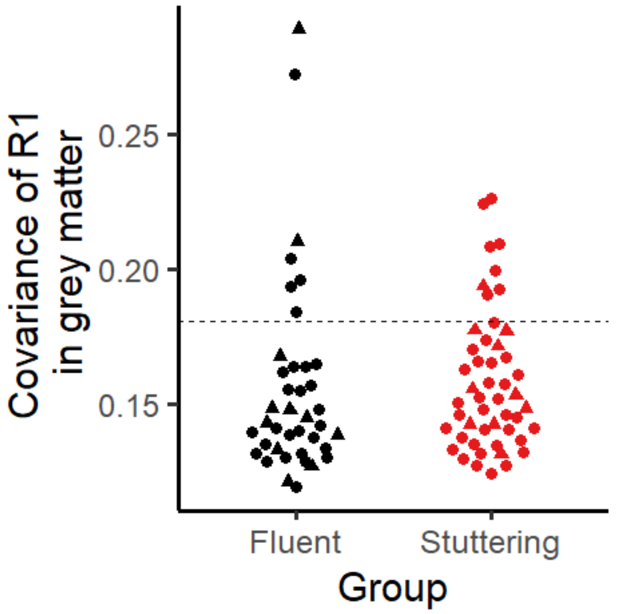

Supplemental Figure 3. Results of semi-automated quality control algorithm. Individuals who are typically fluent shown in black on left; individuals who stutter shown in red on right. Circles are men and triangles are women. Quality control cutoff (dashed horizontal line) was chosen empirically to be consistent between groups and to separate acceptable from unacceptable movement. Only participants with grey matter covariance in R1 map ≤ .18 were included in all subsequent analyses. Data from three additional participants with acquisition artefacts were excluded and not shown here.

Tables

Supplemental Table 1. Parameters for each tissue type across the whole brain.

Parameter	MED (SE) Fluent	MED (SE) Stuttering	Age		Group		Age × group	
			F	*p*	*F*	*p*	*F*	*p*
R1 – white matter	0.9 (.005)	0.9 (.004)	7.4	**0.008**	1.5	0.2	2.2	0.1
R1 – grey matter	0.7 (.003)	0.7 (.002)	15.2	**<0.001**	0.4	0.5	0.0	0.9
MT – white matter	1.6 (.008)	1.5 (.007)	1.1	0.3	0.0	0.9	1.8	0.2
MT – grey matter	0.9 (.004)	0.9 (.003)	9.0	**0.004**	0.8	0.4	0.1	0.8
R2* – white matter	20.9 (.15)	21.0 (.10)	16.6	**<0.001**	0.1	0.8	2.2	0.1
R2* – grey matter	17.8 (.14)	18.0 (.13)	36.0	**<0.001**	3.3	0.1	0.0	0.9
Whole brain volume	1545506 (29660)	1579228 (17909)	0.3	0.6	0.8	0.4	0.4	0.5

Median and standard error for each parameter and tissue type across the whole brain. Differences tested statistically with analyses of variance (R command: aov) with factors of age, group, and age × group

Supplemental Table 2. Effects of age on R2* in statistical clusters; no interaction between age and group

Brain region	Med (SE) Fluent	Med (SE) Stuttering	Age		Group		Age × group	
			F	*p*	*F*	*p*	*F*	*p*
L Superior frontal sulcus	15.7 (0.17)	16.5 (0.13)	25.5	**<0.001**	13.3	**0.001**	0.2	0.7
L Inferior frontal gyrus, *pars opercularis*	16.7 (0.18)	17.4 (0.15)	35.4	**<0.001**	8.3	**0.005**	0.01	0.9
L Inferior frontal sulcus (posterior)	16.5 (0.15)	17.5 (0.16)	32.5	**<0.001**	14.3	**<0.001**	3.5	0.07
L Putamen	17.6 (0.27)	18.8 (0.28)	31.2	**<0.001**	11.7	**0.001**	0.2	0.7
L Frontal operculum / insula	14.9 (.13)	15.8 (.13)	28.8	**<0.001**	23.7	**<0.001**	1.1	0.3
L Precentral gyrus (ventral)	17.4 (0.16)	18.1 (0.15)	41.0	**<0.001**	10.0	**0.002**	0.2	0.7
L Superior parietal lobule	16.2 (0.16)	17.1 (0.18)	19.9	**<0.001**	15.5	**<0.001**	0.1	0.7
L planum temporale	16.2 (0.2)	17.0 (0.19)	11.9	**0.001**	6.5	**0.01**	<0.01	1

Median and standard error (SE) for R2* in grey matter in statistical clusters ($p<.025$) for people who stutter (STUTTERING) and Fluenttrols (FLUENT). Differences tested statistically with analyses of variance (R command: aov) accounting for age, group, and age × group.

Supplemental Table 3. No effect of severity on R2* in individuals who stutter

Brain region	Age		Severity		Age × severity	
	F	*p*	*F*	*p*	*F*	*p*
L Superior frontal sulcus	13.9	**0.001**	0.1	0.7	0.0	1.0
L Inferior frontal gyrus, *pars opercularis*	17.2	**<0.001**	0.9	0.4	0.0	0.9
L Inferior frontal sulcus (posterior)	23.7	**<0.001**	1.2	0.3	0.4	0.5
L Putamen	13.1	**0.001**	0.1	0.7	1.1	0.3
L Frontal operculum / insula	15.8	**<0.001**	2.6	0.1	0.1	0.8
L Precentral gyrus (ventral)	20.1	**<0.001**	3.7	0.06	0.1	0.7
L planum temporale	4.1	**0.05**	0.5	0.5	0.9	0.3
L Superior parietal lobule	7.0	**0.01**	1.0	0.3	0.3	0.6

References

1. Papp D, Callaghan MF, Meyer H, Buckley C, Weiskopf N. Correction of inter-scan motion artifacts in quantitative R1 mapping by accounting for receive coil sensitivity effects. *Magn Reson Med*. 2016;76(5):1478-1485. doi:10.1002/mrm.26058

2. Tabelow K, Balteau E, Ashburner J, et al. hMRI – A toolbox for quantitative MRI in neuroscience and clinical research. *Neuroimage*. 2019;194:191-210. doi:10.1016/j.neuroimage.2019.01.029
